# Aspiration pneumonia in diabetic ketoacidosis: a decade-long analysis of occurrence , associated risk factors and clinical outcomes in 530,700 US hospitalizations

**DOI:** 10.1186/s13098-025-01972-6

**Published:** 2025-10-16

**Authors:** Binbin Tian, Xianghua Cao, Junfen Cheng, Jian Wang, Pengfei Shen, Junde Mo, Guorong Zhong, Guangyuan Zhang

**Affiliations:** 1 Department of Critical Care Medicine, Zhanjiang Central Hospital, Guangdong Medical University, Zhanjiang, 524037 Guangdong China; 2 Department of Anesthesiology, Dongguan Tungwah Hospital, Dongguan, 523110 China; 3 Department of Respiration, The Second Affiliated Hospital of Guangdong Medical University, Zhanjiang, 524003 Guangdong China; 4 Zhanjiang Key Laboratory of Respiratory Chronic Disease Prevention and Control, Guangdong, China; 5 Division of Orthopaedic Surgery, Department of Orthopaedics, Nanfang Hospital, Southern Medical University, Guangzhou, 510515 Guangdong China; 6 General Surgery Department, Nanfang Hospital, Southern Medical University, Guangzhou, 510515 Guangdong China; 7 Department of Emergency, Zhanjiang Central Hospital, Guangdong Medical University, Zhanjiang, 524037 Guangdong China

**Keywords:** Diabetic ketoacidosis, Aspiration pneumonia, Associated risk factors, Nationwide inpatient sample

## Abstract

**Background:**

Aspiration pneumonia (AP) is a serious clinical condition among patients with diabetic ketoacidosis (DKA). This study aimed to determine the occurrence and associated risk factors for AP among patients hospitalized with DKA.

**Methods:**

The National Inpatient Sample (2010–2019) was used to identify adults with DKA. Patients were divided into two groups based on the presence of AP. Multivariate logistic regression was performed to identify risk factors associated with AP, adjusting for demographics, Elixhauser comorbidities, and hospital characteristics.

**Results:**

Among 530,700 hospitalizations for DKA, 2.0% developed AP. Multivariate analysis revealed that age ≥ 45 years, ≥ 2 comorbidities, large hospital of bed size and urban hospital were associated with higher odds of AP. Specific comorbidities (alcohol abuse, congestive heart failure, coagulopathy, drug abuse, fluid and electrolyte disorders, neurological disorders, paralysis, psychoses, weight loss, and dementia) and complications (gastrointestinal hemorrhage, deep vein thrombosis, sepsis, stroke, blood transfusion) were associated with increased risk of AP. Patients with AP were significantly associated with higher hospital mortality (16.9% vs. 1.8%), longer median hospital stays (9 vs. 3 days), increased median healthcare costs ($97,318 vs. $22,835), and higher need for mechanical ventilation (2.2% vs. 0.1%) (*P* < 0.001).

**Conclusion:**

The findings highlight a relatively high occurrence of AP in patients with DKA, strongly associated with various comorbidities. The results emphasize the need for optimized management of high-risk patients to reduce adverse outcomes. Further prospective studies are required to clarify these associations and develop effective prevention strategies.

## Background

Diabetic ketoacidosis (DKA) is a medical emergency characterized by severe metabolic disturbances. Clinical manifestations of DKA range from mild fluid depletion to seizures and coma [1]. Impaired consciousness in DKA, often accompanied by gastroparesis, gastric distension, and vomiting, significantly increases the risk of aspiration [1, 2]. Inhalation of gastric contents into the respiratory tract induces aspiration pneumonia (AP ), resulting in acute chemical inflammation. Clinical presentations vary from subclinical episodes that resolve spontaneously to severe pneumonitis progressing to acute respiratory distress syndrome [3, 4].

Current data indicate that AP constitutes for approximately 5%–15% of community-acquired pneumonia cases [4–6]. Mortality rates for AP exceed those of other pneumonia types by more than twofold [4]. Management of AP frequently requires ICU readmission and prolonged hospitalization, substantially increasing healthcare costs [6]. Extended hospital stays related to AP are also associated with decreased functional capacity, impaired daily activities, and reduced quality of life, particularly in elderly patients [7]. Early identification of patients at increased risk of AP could improve therapeutic interventions and patient outcomes [6].

However, current research has not specifically addressed the risk factors associated with AP in DKA patients. Thus, this study analyzes the occurrence, identifies associated factors, and describes clinical outcomes to fill this critical knowledge gap.

## Methods

### Data source

For this investigation, we employed the Nationwide Inpatient Sample (NIS), which represents the most comprehensive all-payer hospitalization database available in the United States. The NIS is maintained under the Healthcare Cost and Utilization Project (HCUP) by the Agency for Healthcare Research and Quality (AHRQ). It employs a stratified sampling methodology to capture approximately 20% of annual hospital discharges from over 1,000 healthcare facilities across the nation. The sampling frame encompasses more than 97% of the U.S. population [8–10]. The NIS utilizes a complex stratified sampling design based on U.S. census region, hospital ownership, location (urban/rural), teaching status, and bed size to ensure national representativeness. The database provides discharge weights to facilitate the calculation of nationally representative estimates. It includes primary and secondary diagnoses and procedures (coded using ICD-9-CM and ICD-10-CM systems), patient demographics, hospital characteristics, discharge status, and total charges. The data are derived from administrative billing records provided by participating states. AHRQ and HCUP implement rigorous quality control procedures, including systematic validation checks, to ensure data consistency, completeness, and reliability. The validity of ICD codes in the NIS for epidemiological research has been established in numerous previous studies. Given that our research constitutes a retrospective cohort analysis utilizing anonymized, publicly available information from this restricted dataset, it was exempt from Institutional Review Board (IRB) review and ethical clearance requirements, in accordance with ethical principles codified in the Declaration of Helsinki [9, 10].

### Cohort identification and selection criteria

**Fig. 1:**
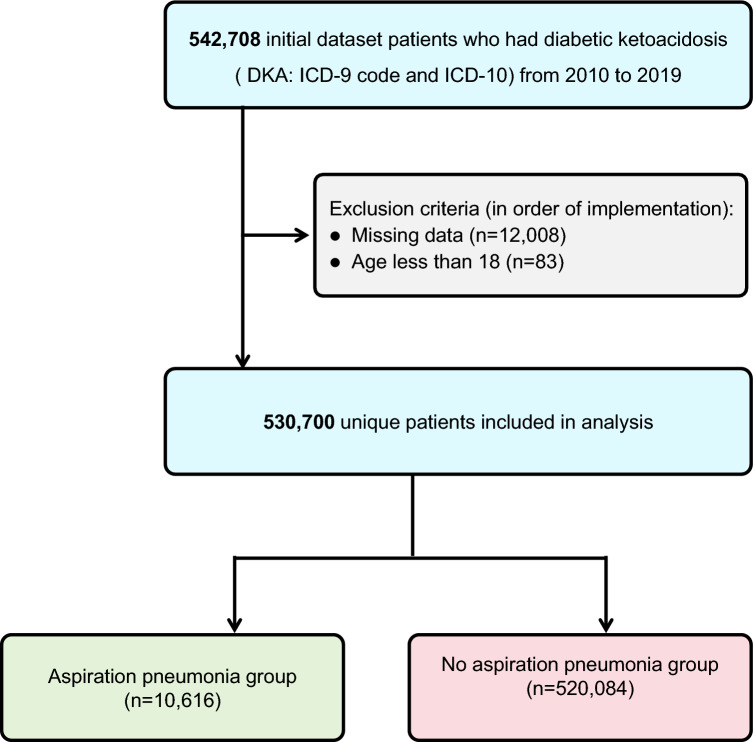
Inclusion/exclusion process of DKA

Our analysis encompassed NIS entries from 2010 through 2019. We identified DKA hospitalizations through diagnostic codes in both ICD-9 and ICD-10 classification systems: for type 1 diabetes with ketoacidosis (250.11, 250.13, E10.1, E10.10, E10.11), type 2 diabetes with ketoacidosis (250.10, 250.12, E11.1, E11.10, E11.11), and additional diabetes variants presenting with ketoacidosis (249.10, 249.11, E08.1, E09.1, E13.1, E13.10, E13.11) [8]. Hospitalizations of AP were recognized using ICD-9-CM code 507.0 and ICD-10-CM code J69.0. These codes specifically denote pneumonitis resulting from the aspiration of food or gastric contents and have been widely validated in epidemiological research based on administrative data [11–14]. Patients younger than 18 years and records with missing critical data were excluded from the analysis (Fig. 1).

The study population was divided according to AP (present vs. absent). Comparative analyses were performed to evaluate demographic characteristics, hospital features, comorbidities, complications, and clinical outcomes between the two groups. Our outcome measures included length of hospital stay, financial burden, mortality, and mechanical ventilation. Comorbidities and complications were identified through predefined diagnostic categories (Table 1).

**Table 1:** Variables used in binary logistic regression analysis

Variables categories	Specific variables
Patient demographics	Age (18–44 years ≤ 64 years and ≥ 65 years), sex (male and female), race (White, Black, Hispanic, Asian or Pacific Islander, Native American and Other)
Hospital characteristics	Type of admission (non-elective, elective), bed size of hospital (small, medium, large), teaching status of hospital (nonteaching, teaching), location of hospital (rural, urban), type of insurance (Medicare, Medicaid, private insurance, self-pay, no charge, other), location of the hospital (northeast, Midwest or north central, south, west)
Commodities	AIDS, alcohol abuse, deficiency anemia, rheumatoid diseases, chronic blood loss anemia, congestive heart failure, chronic pulmonary disease, coagulopathy, depression, diabetes, drug abuse, hypertension, hypothyroidism, liver disease, lymphoma, fluid and electrolyte disorders, metastatic cancer, neurological disorders, obesity, paralysis, peripheral vascular disorders, psychoses, pulmonary circulation disorders, renal failure, solid tumor without metastasis, peptic ulcer disease, valvular disease and weight loss, gastroesophageal reflux disease, dementia
Complications	Arrhythmia, gastrointestinal hemorrhage, deep vein thrombosis, sepsis, stroke, blood transfusion, urinary tract infection

### Statistical analysis

All statistical analyses were conducted using IBM SPSS version 25.0 (Armonk, NY). For comparing continuous variables between groups, independent t-tests were employed, whereas categorical data were examined through chi-square testing. To elucidate factors linked to AP, multivariate logistic regression models were constructed, incorporating demographic characteristics, complications, comorbidities and institutional variables extracted from the NIS database. Results are presented as odds ratios accompanied by their respective 95% confidence intervals. Given our substantial sample population, statistical significance was defined more stringently at *P* < 0.001 [8, 9].

## Results

### Temporal trends and overall Occurrence 

**Fig. 2:**
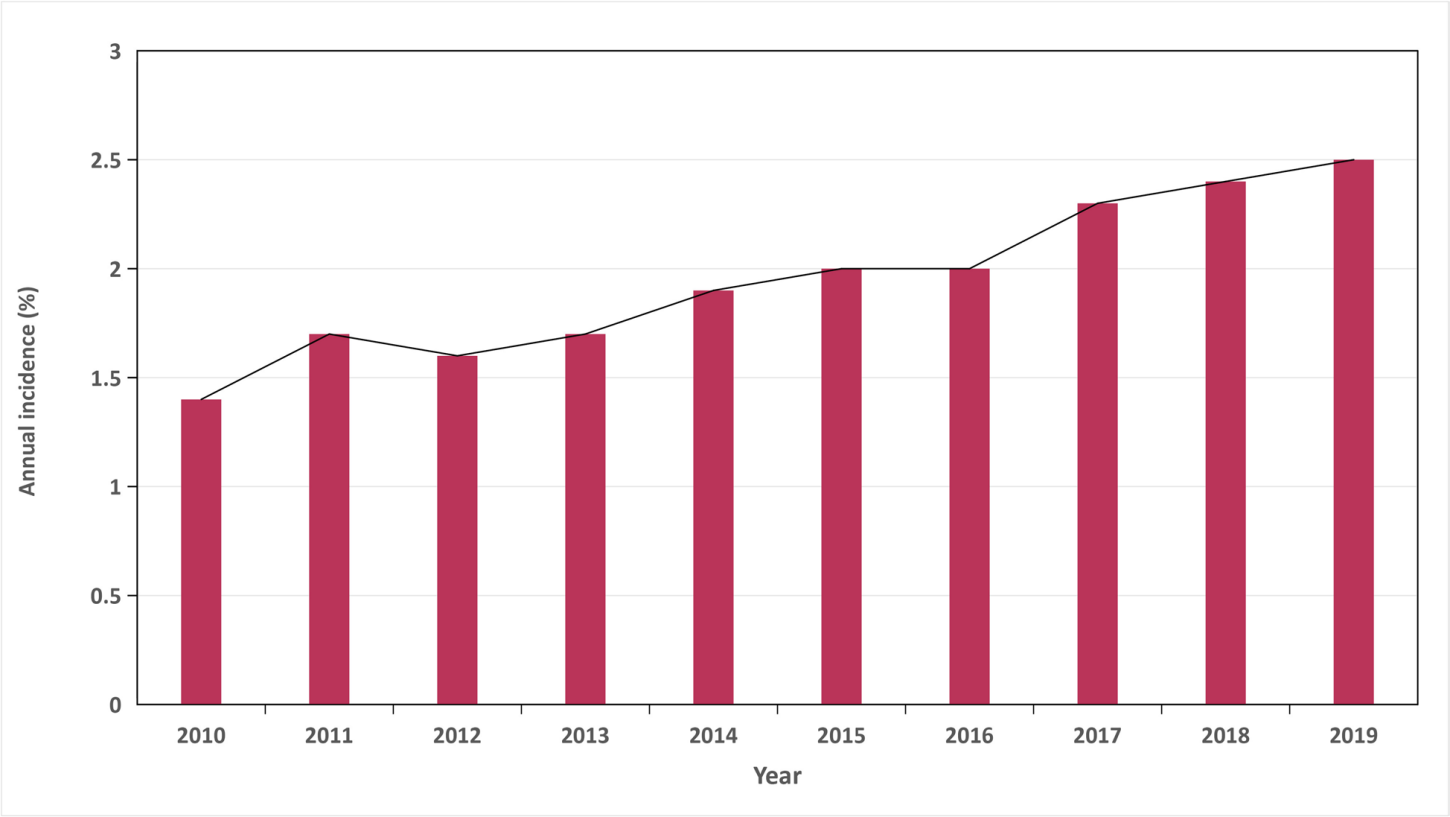
Temporal trends of AP in DKA

Analysis of data from 2010 to 2019 indicated that out of 530,700 patients diagnosed with DKA, 10,616 developed AP, which represents 2.0% of the total hospitalizations. A noticeable upward trend in the occurrence of AP was recorded, increasing from 1.4% in 2010 to 2.5% by 2019 (Fig. 2).

### Patient demographics

The median age in the cohort with AP was significantly greater than that of the non-AP group, with ages recorded at 57 years and 40 years, respectively (*P* < 0.001). Demographic analysis revealed that middle-aged adults (those aged 45–64 years) represented a larger percentage of the AP population compared to their non-AP counterparts, at 42.1% vs. 33.5% (*P* < 0.001). Conversely, younger adults (aged 18–44 years) were less frequently represented in the AP cohort, accounting for 23.7% in contrast to 53.2% in the non-AP group (*P* < 0.001). Additionally, racial analysis indicated a higher prevalence of AP among White patients, with rates of 61.2% compared to 55.0% among the non-AP group. In contrast, lower rates were noted in Black patients (17.9% vs. 23.6%) and Hispanic patients (10.0% vs. 11.8%) (*P* < 0.001). Furthermore, patients diagnosed with AP exhibited a greater occurrence of comorbidities, with 90.7% managing three or more comorbidities, as opposed to 56.0% within the non-AP group (*P* < 0.001) (Table 2).

**Table 2:** Patient characteristics and outcomes with DKA (2010–2019)

Characteristics	AP	No AP	P
Total (n = count)	10,616	520,084	
Total occurrence (%)	2.0		
Age (median, years)	57 (45,69)	40 (25,55)	< 0.001
Age group (%)			
18–44	2,503 (23.7%)	253,599 (53.2%)	< 0.001
45–64	4,459 (42.1%)	159,852 (33.5%)	
65–74	1,885 (17.8%)	39,492 (8.3%)	
≥ 75	1,735 (16.4%)	24,038 (5.0%)	
Gender			
Male	5,959 (56.1%)	257,896 (49.6%)	< 0.001
Female	4,657 (43.9%)	262,188 (50.4%)	
Race (%)			
White	6,595 (61.2%)	285,896 (55.0%)	< 0.001
Black	1,902 (17.9%)	122,964 (23.6%)	
Hispanic	1,059 (10.0%)	61,151 (11.8%)	
Asian or Pacific Islander	269 (2.5%)	6,677 (1.3%)	
Native American	92 (0.9%)	4,891 (0.9%)	
Other	799 (7.5%)	38,505 (7.4%)	
Number of Comorbidity (%)			
0	31 (0.3%)	32,047 (6.2%)	< 0.001
1	253 (2.4%)	91,473 (17.6%)	
2	707 (6.7%)	105,066 (20.2%)	
≥ 3	9,625 (90.7%)	291,498 (56.0%)	
Type of insure (%)			
Medicare	5,093 (48.0%)	127,542 (24.5%)	< 0.001
Medicaid	2,405 (22.7%)	159,911 (30.7%)	
Private insurance	1,952 (18.4%)	142,005 (27.6%)	
Self-pay	777 (7.3%)	65,724 (12.6%)	
No charge	64 (0.6%)	5,303 (1.0%)	
Other	325 (3.1%)	19,599 (3.8%)	
Bed size of hospital (%)			
Small	1,641 (15.5%)	95,489 (18.4%)	< 0.001
Medium	3,119 (29.4%)	148,987 (28.6%)	
Large	5,856 (55.2%)	275,599 (53.0%)	
Region of hospital (%)			
Northeast	1,870 (17.6%)	79,779 (15.3%)	< 0.001
Midwest or North Central	2,155 (20.3%)	111,767 (21.5%)	
South	4,240 (39.9%)	225,985 (43.5%)	
West	2,351 (22.1%)	102,553 (19.7%)	
Elective admission (%)	338 (3.2%)	15,979 (3.1%)	0.510
Type of hospital (teaching %)	6,779 (63.9%)	312,780 (60.1%)	< 0.001
Location of hospital (urban, %)	9,851 (92.8%)	45,9831 (88.4%)	< 0.001
Died (%)	1,792 (16.9%)	9,262 (1.8%)	< 0.001
LOS (median, d)	9 (5–16)	3 (2–5)	< 0.001
TOTCHG (median, $)	97,318 (49,342–189,848)	22,835 (13,347–42,496)	< 0.001
Mechanical ventilation (%)	231 (2.2%)	633 (0.1%)	< 0.001

AP: aspiration pneumonia; LOS: Length of stay ;TOTCHG: Total charge

### Hospital characteristics

Data from hospitals revealed that AP was more frequently diagnosed in larger bed size of hospitals (55.2% vs. 53.0%), urban hospitals (92.8% vs. 88.4%), and teaching hospitals (63.9% vs. 60.1%) (*P* < 0.001). Regionally, the West had the highest percentage of AP hospitalizations (22.1% vs. 19.7%, *P* < 0.001), followed closely by the Northeast (17.6% vs. 15.3%, *P* < 0.001). Regarding type of insurance, Medicare accounted for a significantly larger share of AP hospitalizations (48.0% vs. 24.5%). Conversely, Medicaid (22.7% vs. 30.7%), private insurance (18.4% vs. 27.6%), and self-pay patients (7.3% vs. 12.6%) were found to be less common among the AP cohort (*P* < 0.001) (Table 2).

### Health outcomes related to AP

AP was associated with significantly worse clinical outcomes. These outcomes include extended median hospital stays (9 days compared to 3 days), higher median treatment costs ($97,318 vs. $22,835), elevated mortality rates (16.9% vs. 1.8%), and an increased necessity for mechanical ventilation (2.2% vs. 0.1%) (*P* < 0.001) (Table 2).

###  Associated factors for AP identified through multivariate analysis

**Fig. 3:**
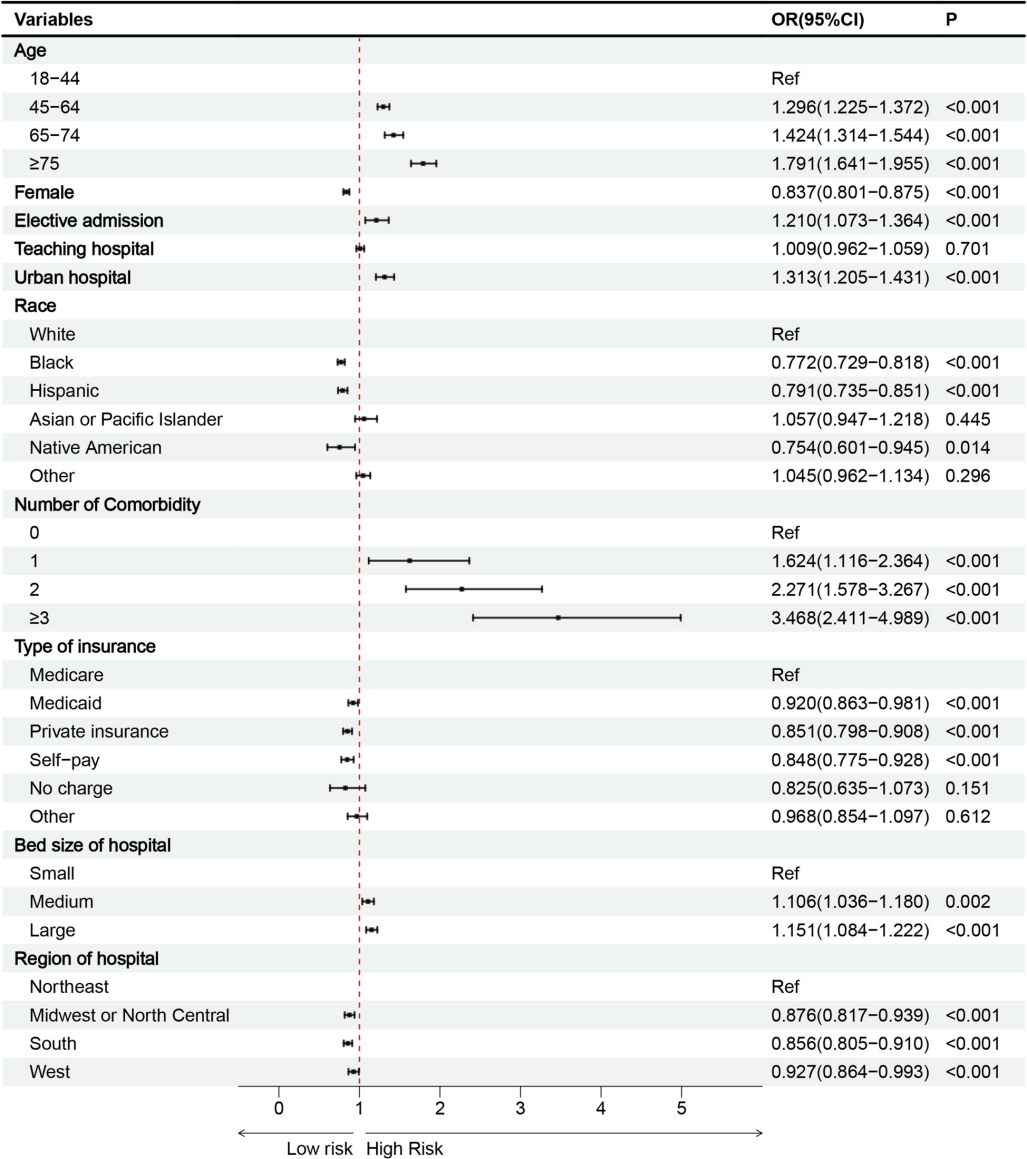
Risk factors associated with AP in DKA

Multivariate analysis has identified several important predictors of AP. These predictors include age ≥ 45 years, comorbidity ≥ 2, large bed size of hospitals and urban hospitals (Fig. 3).

**Fig. 4:**
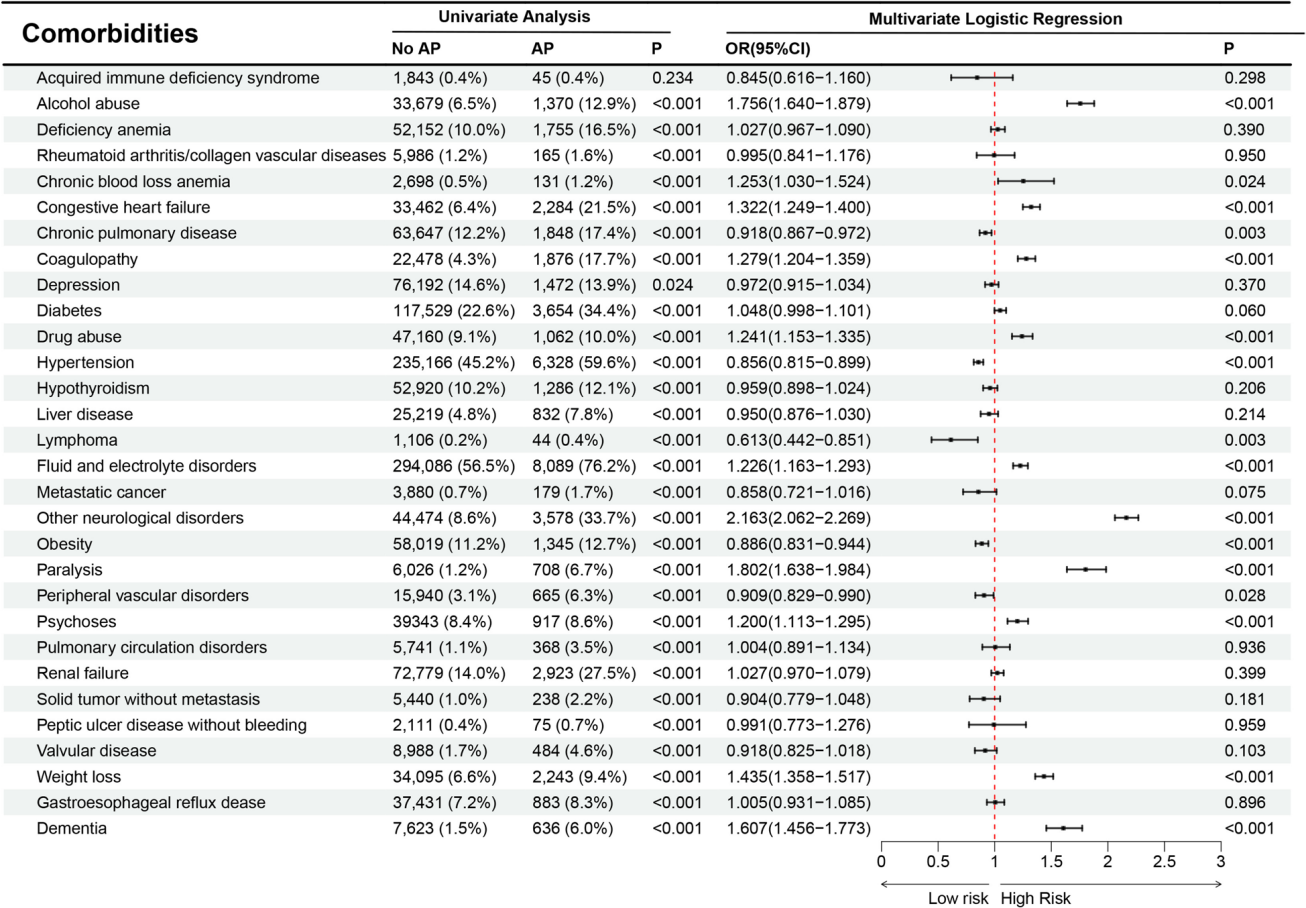
Relationship between AP and comorbidities

Significant comorbidities identified as associated risk factors for AP included alcohol abuse, congestive heart failure, coagulopathy, drug abuse, fluid and electrolyte disorders, other neurological disorders, paralysis, psychoses, weight loss, and dementia (Fig. 4).

Conversely, several factors are associated with a reduced risk of AP. These factors include female, Black/Hispanic, private insurance/self-pay, and hospitals in the Midwest/North Central, South, and West regions (Fig. 3).

### Clinical complications associated with AP during DKA

**Fig. 5:**
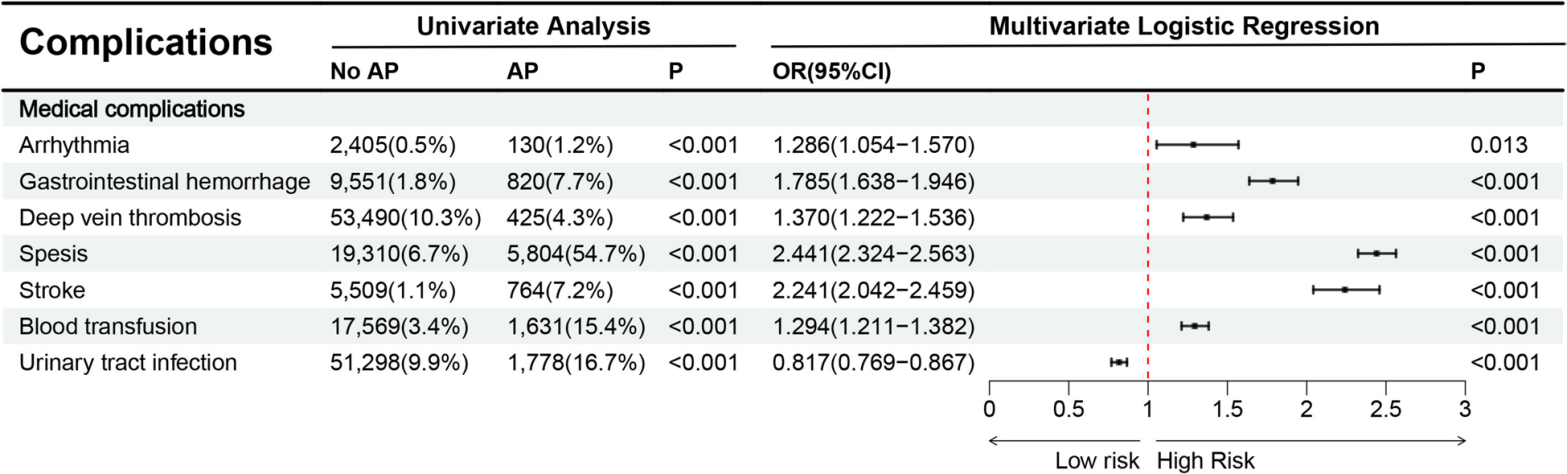
Relationship between AP and complications

A multivariate analysis revealed several medical complications linked to AP, including gastrointestinal hemorrhage, deep vein thrombosis, sepsis, stroke, blood transfusion (Fig. 5).

## Discussion

### Principal findings

The main findings of the present study are as follows: The overall occurrence of AP in DKA hospitalizations, derived from the NIS database, was 2.0% over the last decade, representing approximately 530,000 admissions. Importantly, the occurrence of AP gradually increased from 1.4% in 2010 to 2.5% in 2019. Hospitalized DKA patients who developed AP carried a substantial healthcare burden with significantly increased mortality (16.9%). Multivariate analysis revealed multiple factors linked to AP, including blood transfusion, alcohol abuse, congestive heart failure, coagulopathy, drug abuse, fluid and electrolyte disorders, additional neurological disorders, paralysis, psychosis, weight loss, dementia, gastrointestinal hemorrhage, deep vein thrombosis, sepsis, and stroke. This investigation represents the first and largest longitudinal study on AP in DKA patients within a U.S. cohort to date.

###  Occurrence and mortality

Analysis of nationally representative data (2010–2019) showed an occurrence of 2.0% for AP among patients hospitalized for DKA, with significantly elevated mortality (16.9%) in affected individuals. The observed occurrence broadly consistent with results from Patel et al., who reported a 1.9% occurrence of AP in 1.8 million hospitalized esophagogastroduodenoscopy patients [15]. Using the Multiple Cause of Death Dataset (1999–2017), Gupte et al. identified 1,112,944 cumulative deaths due to AP (annual mean: 58,576 deaths), accounting for 30.1% of all pneumonia-related fatalities [14]. AP represents a critical medical condition that can trigger severe clinical deterioration, including death [14].

The occurrence of AP demonstrated a steady increase (1.4% to 2.5%) from 2010 to 2019. The mechanisms underlying this increase remain unclear but are likely multifactorial. Recent studies report comparable epidemiological trends, with increased hospital admissions for DKA as one plausible explanation [16]**.** The observed increase may reflect improvements in clinical detection of AP and enhanced identification of severe presentations previously overlooked [17–19]. Since 2000, institutional acceptance of palliative care directives has progressively increased. Strong evidence suggests these directives significantly reduce intensive therapeutic interventions and terminal hospitalizations [14, 20].

AP was associated with substantial negative clinical outcomes, including prolonged median hospitalization (9 days vs. 3 days) and increased median treatment costs ($97,318 vs. $22,835). Previous literature consistently demonstrates that patients with AP require longer hospital stays compared to non-AP patients [21, 22]. These findings are consistent with our observational data. Additionally, elderly patients recovering from pneumonia typically experience longer hospital stays and slower recovery, significantly increasing healthcare expenditures [23–25]. Comparative analyses indicate that treatment costs for AP exceed those of conventional pneumonia by approximately fivefold. Early diagnosis combined with preventive strategies can substantially reduce financial burdens, improve patient prognosis, and enhance the overall quality of healthcare services [26].

### Associated factors

Previous studies have established clear associations between conditions causing dysphagia, severe impairment of consciousness, and reduced capacity for secretion clearance [14, 27, 28]. The current analysis confirms advanced age as a factor significantly associated with AP. This relationship may involve two primary mechanisms: impaired mucociliary clearance and inadequate oral hygiene maintenance. A documented decline in the swallowing reflex with aging may further explain increased susceptibility among elderly individuals [29]. Aging is also associated with gradual impairment of immune responses [30] and increased TNF-α expression, which negatively regulates T-cell-mediated immunity [31]. Elderly individuals experiencing decreased mobility, cognitive dysfunction, and dementia may have an increased risk of pulmonary aspiration following diagnostic or therapeutic endoscopy [32, 33]. A comprehensive meta-analysis involving 21 studies of geriatric patients with frailty syndrome identified additional independent risk factors, including antipsychotic medications, proton pump inhibitor use, and poor oral health [34].

The present study identified a higher prevalence of comorbid conditions in patients with AP. Previous research has consistently reported associated conditions such as fibrotic lung disease, bronchogenic carcinoma, congestive cardiac failure, end-stage renal disease, prior stroke, dementia, psychiatric disorders, low body weight, enteral tube feeding, and various cardiac arrhythmias [22, 33, 35, 36]. Additional associations include sepsis and fluid and electrolyte disturbances [33, 35].

Interestingly, a reduced risk of AP was observed in female patients, possibly related to estrogen effects. Dai et al. demonstrated that estrogen exerts cell type-specific modulation of microRNA expression in immune cells, suggesting a regulatory role in estrogen-mediated immunomodulation [37]. Another explanatory factor may involve higher tobacco use, alcohol consumption, and behaviors increasing aspiration risk among male patients [38]. Reduced risk of AP was associated with non-Caucasian ethnicity, private insurance or self-funded care, and specific regional hospital patterns. Ethnic differences may be partly explained by healthcare access inequalities, potentially correlate with underdiagnosis in underserved populations [39]. Socioeconomic disparities may also influence healthcare-seeking behaviors, with patients from higher socioeconomic groups likely receiving earlier medical attention, thus preventing disease progression [40]. Significant geographic variation exists in the application of preventive measures against pulmonary aspiration across U.S. medical centers [41, 42].

### Clinical and research implications

The relative rarity of AP contributes to limited recognition among clinicians, particularly resident physicians. Diagnostic difficulty arises from phenotypic overlap between AP and common pulmonary syndromes, leading to delayed diagnosis and management [42–44]. This study contributes important insights into the literature regarding risk factors and survival outcomes in AP. Given the high mortality rates, implementing structured educational programs involving simulated clinical scenarios is necessary to standardize diagnostic and therapeutic approaches.

### Limitations

Several inherent limitations exist in this study. First, the identification of AP relied on clinician diagnosis, typically involving exclusion of other etiologies [45]. Consequently, the true occurrence of AP might be underestimated. Second, as the NIS is an administrative database structured around hospitalizations rather than individual patients, it was not possible to identify or exclude individuals with multiple DKA hospitalizations during the study period. This may result in overrepresentation of patients with recurrent, severe, or poorly controlled diabetes and restricts the ability to track patients longitudinally across multiple hospital admissions. Third, using diagnostic codes for cohort identification introduces potential misclassification bias, a common limitation of large-scale administrative data research. Systematic coding biases, including limitations in diagnostic codes and variability between different ICD systems, may contribute to underestimation or misclassification of disease burden [11]. Fourth, the NIS lacks detailed laboratory parameters (e.g., pH, bicarbonate, anion gap) and comprehensive pharmacologic information, particularly regarding preadmission outpatient medication use (e.g., Sodium-Glucose Cotransporter 2 (SGLT2) inhibitors) or insulin adherence. This limitation precluded adjustment for DKA severity-a potential unmeasured confounder-and restricted our ability to evaluate pharmacological contributors to AP risk. Nevertheless, significant strengths counterbalance these limitations, notably the extensive inpatient population and elimination of regional bias through comprehensive national sampling [15]. Finally, due to the cross-sectional nature of the NIS, the temporal relationship between DKA and AP could not be definitively established. Although AP may precipitate DKA due to infectious stress, DKA itself may increase AP risk through mechanisms such as impaired mental status, gastroparesis, and vomiting. Thus, the direction of causality remains uncertain. Future prospective studies incorporating longitudinal biochemical and detailed medication records are warranted to better elucidate causal relationships.

## Conclusion

The current cohort study confirms that AP is a clinically significant, though relatively uncommon complication, associated with notably high mortality, prolonged hospitalization, and substantial economic burden. Risk-factor analysis identified significant associations between AP and factors including male sex, advanced age, and comorbidities. These findings emphasize the need for improved risk assessment and protocol optimization for high-risk patients, while prospective studies are needed to validate these associations and guide evidence-based prevention strategies.

## Data Availability

This study is based on data provided by Nationwide Inpatient Sample (NIS) database, part of the Healthcare Cost and Utilization Project, Agency for Healthcare Research and Quality. The NIS database is a large publicly available full-payer inpatient care database in the United States and the direct web link to the database is https://www.ahrq.gov/data/hcup/index.html. Therefore, individual or grouped data cannot be shared by the authors.

## Abbreviations

AP: Aspiration Pneumonia
DKA: Diabetic Ketoacidosis
NIS: Nationwide Inpatient Sample
ICD-9-CM: International Classification of Diseases, Ninth Revision, Clinical Modification
ICD-10-CM: International Classification of Diseases, Tenth Revision, Clinical Modification
